# Volatile anesthetics maintain tidal volume and minute ventilation to a greater degree than propofol under spontaneous respiration

**DOI:** 10.1186/s12871-021-01438-y

**Published:** 2021-10-06

**Authors:** Xuechao Hao, Mengchan Ou, Yu Li, Cheng Zhou

**Affiliations:** 1 Departments of Anesthesiology, West China Hospital of Sichuan University, Chengdu, 610041 China; 2 Laboratory of Anesthesia & Critical Care Medicine, Translational Neuroscience Center, The Research Units of West China-Chinese Academy of Medical Sciences, West China Hospital of Sichuan University, Chengdu, 610041 China

**Keywords:** General Anesthetics, Spontaneous Respiration, Whole-body Plethysmograph

## Abstract

**Background:**

Although general anesthetics depress spontaneous respiration, the comprehensive effect of general anesthetics on respiratory function remains unclear. We aimed to investigate the effects of general anesthetics on spontaneous respiration in non-intubated mice with different types and doses of general anesthetic.

**Methods:**

Adult C57BL/6 J mice were administered intravenous anesthetics, including propofol and etomidate, and inhalational anesthetics, including sevoflurane and isoflurane in vivo at doses of 0.5-, 1.0-, and 2.0-times the minimum alveolar concentration (MAC)/median effective dose (ED_50_) to induce loss of the righting reflex (LORR). Whole-body plethysmography (WBP) was applied to measure parameters of respiration under unrestricted conditions without endotracheal intubation. The alteration in respiratory sensitivity to carbon dioxide (CO_2_) under general anesthesia was also determined. The following respiratory parameters were continuously recorded during anesthesia or CO_2_ exposure: respiratory frequency (FR), tidal volume (TV), minute ventilation (MV), expiratory time (TE), inspiratory time (TI), and inspiratory–expiratory time ratio (I/E), and peak inspiratory flow.

**Results:**

Sub-anesthetic concentrations (0.5 MAC) of sevoflurane or isoflurane increased FR, TV, and MV. With isoflurane and sevoflurane exposure, the CO_2_-evoked increases in FR, TV, and MV were decreased. Compared with inhalational anesthetics, propofol and etomidate induced respiratory suppression, affecting FR, TV, and MV. In 100% oxygen (O_2_), FR in the group that received propofol 1.0-times the ED_50_ was 69.63 ± 33.44 breaths/min compared with 155.68 ± 64.42 breaths/min in the etomidate-treated group. In the same groups, FR was 88.72 ± 34.51 breaths/min and 225.10 ± 59.82 breaths/min, respectively, in 3% CO_2_ and 144.17 ± 63.25 breaths/min and 197.70 ± 41.93 breaths/min, respectively, in 5% CO_2_. A higher CO_2_ sensitivity was found in etomidate-treated mice compared with propofol-treated mice. In addition, propofol induced a greater decrease in FR, MV, and I/E ratio compared with etomidate, sevoflurane, and isoflurane at equivalent doses (all *P* < 0.05).

**Conclusions:**

General anesthetics differentially modulate spontaneous breathing in vivo. Volatile anesthetics increase FR, TV, and MV at sub-anesthetic concentrations, while they decrease FR at higher concentrations. Propofol consistently depressed respiratory parameters to a greater degree than etomidate.

## Background

More than 300 million major surgical procedures requiring general anesthesia or analgesics are conducted worldwide each year [1, 2]. General anesthetics and analgesics induce respiratory depression, which is a critical issue in clinical practice, especially for sedative procedures requiring maintenance of spontaneous respiration [3]. Multiple studies have explored the depressant effect of opioids on respiration, but the effects of general anesthetics are not well elucidated [4, 5].

General anesthetics exert various clinically important actions, including hypnosis, amnesia, and immobility [6], as well as respiratory disturbance. Even though a depressant effect is a commonly suggested effect of general anesthetics, significant differences are observed in respiratory behavior between general anesthetics. Propofol depresses ventilation by affecting central chemoreceptor sensitivity, reducing the ventilatory response to hypercapnia, and reducing the ventilatory adaptation to hypoxia, even at sub-anesthetic doses [7, 8]. During dexmedetomidine infusion, respiratory frequency (FR) is significantly increased, and the overall apnea/hypopnea index is significantly decreased [9].

Volatile anesthetics, including isoflurane and sevoflurane, are preferred over intravenous anesthetics, such as propofol and etomidate, in conditions necessitating maintenance of spontaneous respiration, such as anticipated difficulties with endotracheal intubation. Both volatile anesthetics and intravenous anesthetics decrease FR, tidal volume (TV), and minute ventilation (MV) [10–13]. However, early clinical studies with small sample sizes indicated that volatile anesthetics, including enflurane, isoflurane, and sevoflurane, increase FR [10, 14]. Multiple structures associated with respiration are affected by general anesthetics, including the ventral medulla, the retrotrapezoid nucleus (RTN), and phrenic motor neurons [13, 15]. Uncovering the respiratory-related response in vivo during exposure to general anesthetics may help to identify the underlying mechanism. However, limitations exist in previous studies, including restricted study doses and time courses.

As a physical stimulant, carbon dioxide (CO_2_) plays an important role in modulation of respiration by general anesthetics. Studies revealed an increase in the partial pressure of CO_2_ in artery (PaCO_2_) after exposure to general anesthetics. The increase in FR under volatile anesthesia is considered to be a compensatory effect resulting from an elevated PaCO_2_ and respiratory depression [10, 14, 16]. However, lack of an increase in FR was found during intravenous anesthesia. Differences in the manipulation of neuronal processes that are sensitive to CO_2_ might contribute to the discrepancy in respiratory responses induced by volatile versus intravenous anesthetics. Even so, the effect of general anesthetics on respiratory responses to CO_2_ remains unclear.

Inhaled and intravenous anesthesia are two most common approach in clinic. In this study, we chose these four classic drugs of inhaled (sevoflurane and isoflurane) and intravenous anesthetics (propofol and etomidate). The dose-related and time-related effects of the four general anesthetics on respiratory behaviors in mice were explored using whole-body plethysmography (WBP). The respiratory responses to CO_2_ during exposure to general anesthetics were also explored. The results of this study may encourage further research into the mechanisms underlying modulation of respiration by general anesthetics.

## Methods

### Animals

All protocols were approved by the Institutional Animal Experimental Ethics Committee of Sichuan University (Chengdu, Sichuan, China) in accordance with the animal care guidelines of the National Institutes of Health. Endeavors were made to minimize suffering and to reduce the number of mice used.

Experiments were performed in wild type C57BL/6 J male mice aged 12 weeks, weighing 20–25 g. All mice were housed in standard conditions, with a 12-h light/dark cycle and with ad-libitum access to food and water. All experiments were performed during the light cycle (from 9:00 am to 5:00 pm).

### Whole-body Plethysmograph

Whole-body Plethysmograph (Buxco FinePointe Series WBP 4-site system, Data Sciences International, New Brighton, MN, USA) offers a precise, non-invasive, quantitative approach to measure respiratory parameters in conscious, freely moving animals. The system relies on a specially designed chamber in which the subject is placed and allowed to breathe freely under natural conditions, unrestrained and untethered.

The volume of the plethysmography chamber was 480 ml. A constant flow was driven by an oxygen (O_2_) cylinder connected to the chamber, which ensured continuous flow at 0.5 ± 0.1 L/min of gas, thereby preventing CO_2_ accumulation. Hyperoxia and hypercapnia and/or volatile anesthetics were continuously induced/administered into the chamber through the flow pump.

FinePointe software was used to analyze incoming data and create instant reports. The following respiratory parameters were registered: FR, TV, MV, peak inspiratory flow, inspiratory time (Ti), and expiratory time (Te). Except for FR, all measured respiratory parameters (including TV and MV) were normalized to body weight to make them comparable between mice with different body weights. One technician who was blinded to the animal groups measured the respiratory outputs in vivo, and another researcher analyzed the data.

### General anesthetics and CO_2_ administration

Two volatile anesthetics (sevoflurane and isoflurane) and two intravenous anesthetics (propofol and etomidate) were used in this study. According to our previous studies and studies of others [17, 18], equipotent doses for inducing loss of the righting reflex (LORR) (minimum alveolar concentration [MAC]/median effective dose [ED_50_]) of sevoflurane, isoflurane, propofol, and etomidate were 1.58%, 0.86%, 70 mg/kg (intraperitoneally [i.p.]), and 8.85 mg/kg (i.p.), respectively. We used doses of 0.5-, 1.0-, and 2.0-times MAC/ED_50_ required to induce LORR. Propofol at doses of 70 mg/kg, and 140 mg/kg, and etomidate at doses of 8.85 mg/kg, and 17.7 mg/kg, were injected i.p., respectively. For inhalational anesthetics, different concentrations of sevoflurane (0.63%, 1.58%, and 3.16%) and isoflurane (0.34%, 0.86%, and 1.72%) were delivered. A sample size of 8–10 mice for each dose or concentration was used, and all mice were used only once.

Propofol and etomidate (Fresenius Kabi, Bad Homburg, Germany) were injected i.p. All mice received a similar injected volume to exclude the effects of volume variation on respiratory depression in the propofol and etomidate groups.

For sevoflurane (Abbott Pharmacology Ltd., Co., Shanghai, China) and isoflurane (North Chicago, IL, USA) administration, mice were kept in the plethysmography chamber, which comprised a gas inlet and outlet. Sevoflurane or isoflurane was applied into the chamber through the inlet, with a continuous flow of 100% O_2_ at a rate of 0.5 ± 0.1 L/min. Concentrations of sevoflurane and isoflurane were monitored in real-time using the RGM monitor (Datex-Ohmeda, Louisville, CO, USA). For control mice, the chamber was filled with 100% O_2_ at a flow rate of 0.5 L/min.

As indicated by our preliminary experiments, the concentration of sevoflurane or isoflurane in the chamber was balanced after 5 min of delivery, which was detected at the outlet. Drug washout was achieved by suctioning the chamber and flowing fresh air into the chamber between each experiment.

### Behavior test

On the day of the experiment, mice were transported to the laboratory at least 2 h before the start of the experiment. Prior to recording, mice were placed into the plethysmography chamber with no restriction for a minimum of 2 h to allow acclimatization. Mice were awake and calm during testing. Over-excited mice with a high level of locomotor activity or environmental exploration were excluded from anesthetic treatment and behavioral testing.

For control mice, 100% O_2_ was applied for 30 min. For mice exposed to anesthetic, different doses of sevoflurane, isoflurane, propofol, and etomidate (as mentioned above) were applied, respectively, with 100% O_2_ for 30 min. Then, the hyperoxic–hypercapnic experiment was conducted with gas mixtures of 3% CO_2_, 5% CO_2_, and 7% CO_2_ balanced with 100% O_2_ (0.5 L/min). In the time-course experiments, mice were administered three anesthetics with 100% O_2_ (1 L/min) for 30 min.

All experiments were performed at room temperature (22 °C ± 1.5 °C) and humidity (58% ± 5%). Calibration of the plethysmography chamber system was performed once per day before the experiment according to WBP instructions. After each recording, the chamber was cleaned thoroughly with 75% ethanol.

During the experiments, raw respiratory parameters were continuously recorded, and average values were calculated every 2 s. The behavioral state of mice was classified as immobile, exploring, grooming, or undefined. In practice, only data recorded during immobility was considered in the comparison, at least 15 min after propofol or volatile anesthetic delivery. Treatments and tests were conducted randomly on mice in different groups to preclude potential confounding factors. The behavioral test and respiratory parameter analysis were performed with independent researchers who were blinded to the study aims and protocol.

### Statistical analysis

The distribution of values in each set of experiments was tested for normality using the D’Agostino-Pearson omnibus test or the Shapiro–Wilk test. Values are expressed as mean ± standard deviation. The means among groups were compared with with one -way or two-way analysis of variance followed by Bonferroni correction for post-hoc analysis. Differences among groups were considered statistically significant when *P* was < 0.05. Statistical analyses were performed using Prism 7.0 software (GraphPad Software, San Diego, CA, USA) and IBM SPSS Statistics 22.0 (IBM Corp., NY, USA).

## Results

### Sub-anesthetic concentrations of volatile anesthetics increase FR, TV, and MV in wild type mice

**Fig. 1:**
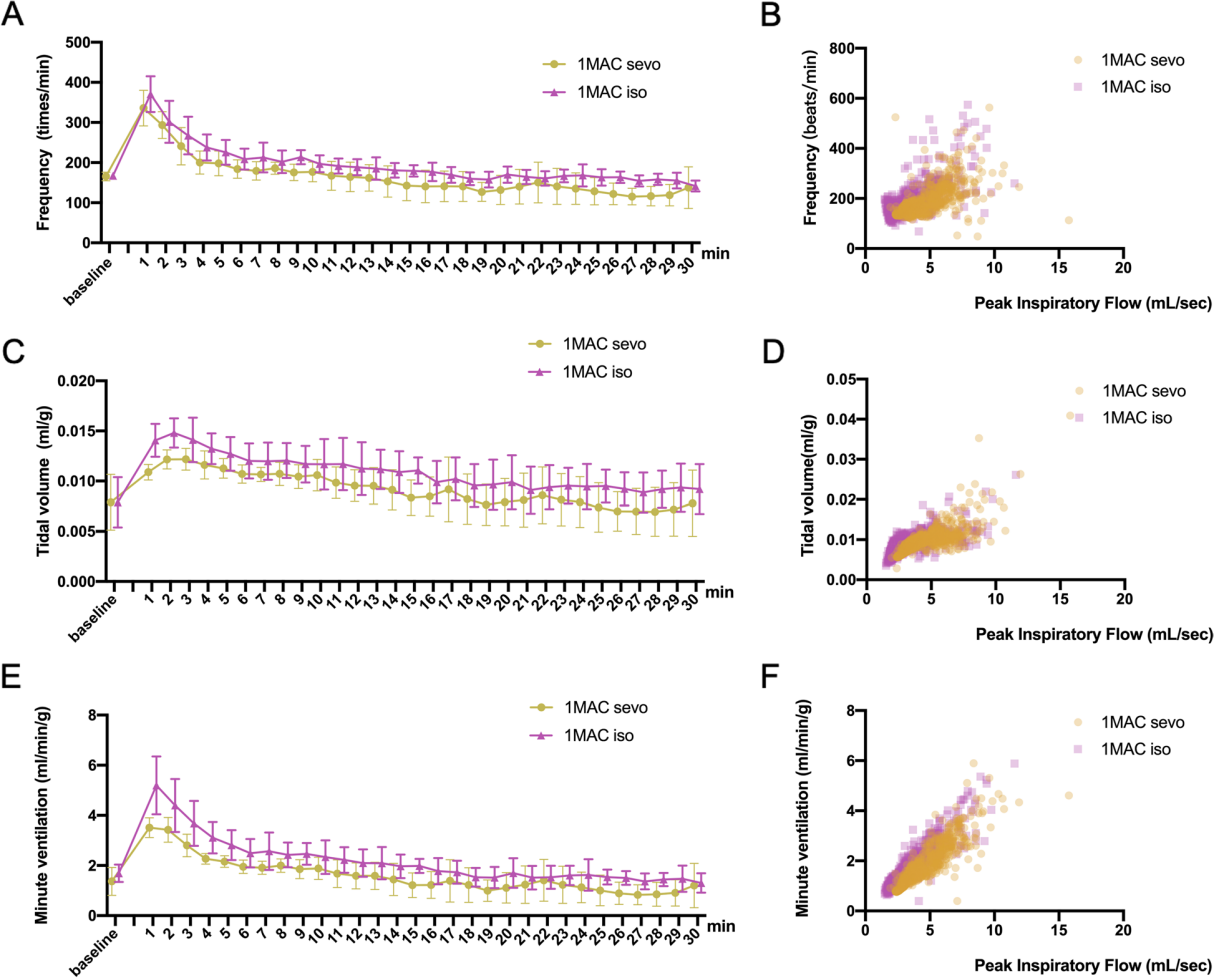
Comparison between 1 minimum alveolar concentration (MAC) sevoflurane and isoflurane. **A** Time-course study of respiratory frequency with 1 MAC sevoflurane and isoflurane. **B** Scatterplot of respiratory frequency versus airflow (ml/sec) for each breath (dot) taken during the 30-min recording. **C** Time-course study of tidal volume with 1 MAC sevoflurane and isoflurane. **D** Scatterplot of tidal volume versus airflow (ml/sec) for each breath (dot) taken during the 30-min recording. **E** Time-course study of minute ventilation with 1 MAC sevoflurane and isoflurane. **F** Scatterplot of minute ventilation versus airflow (ml/sec) for each breath (dot) taken during the 30-min recording. Data are expressed as mean ± standard deviation

There were no measurable differences in respiratory activity among all experimental mice when exposed to room air, 100% O_2_, 3% CO_2_, and 5% CO_2_ conditions (data not shown). During the comparison of inhalational anesthetics, mice had similar FR, TV, and MV values at baseline (100% O_2_). Mice showed a reactive increase in FR, TV, and MV when exposed to 1 MAC sevoflurane and isoflurane with 100% O_2_ at the beginning. Mice stabilized after 10–12 min of recording. The timeline with 1 MAC of sevoflurane and isoflurane are presented in (Fig. 1 A-F). There was no difference between two groups in FR, TV and MV.

**Fig. 2:**
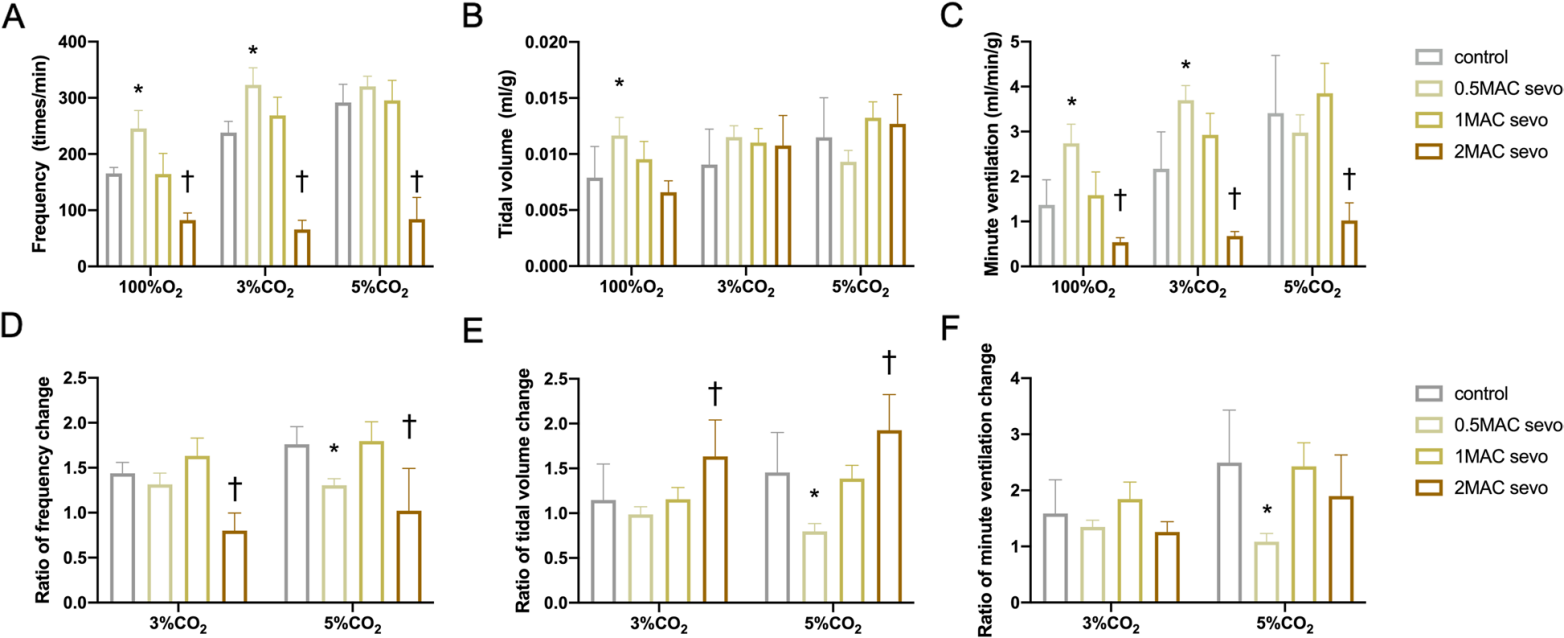
Effect of sevoflurane on respiratory function in a dose-dependent manner in vivo. **A-C** Change in respiratory frequency (**A**), tidal volume (**B**), and minute ventilation (**C**) in the control group and with three doses of sevoflurane (0.5 MAC, 1 MAC, and 2 MAC) are depicted. **D-F** Carbon dioxide induced change ratios in respiratory frequency (**D**), tidal volume (**E**), and minute ventilation (**F**) in the control group and with three doses of sevoflurane (0.5 MAC, 1 MAC, and 2 MAC) are depicted. Data are expressed as mean ± standard deviation. *, #, † indicate that there were significant differences compared with control in 0.5 MAC, 1 MAC, and 2 MAC groups, respectively, by two-way ANOVA (*P* < 0.05)

**Fig. 3:**
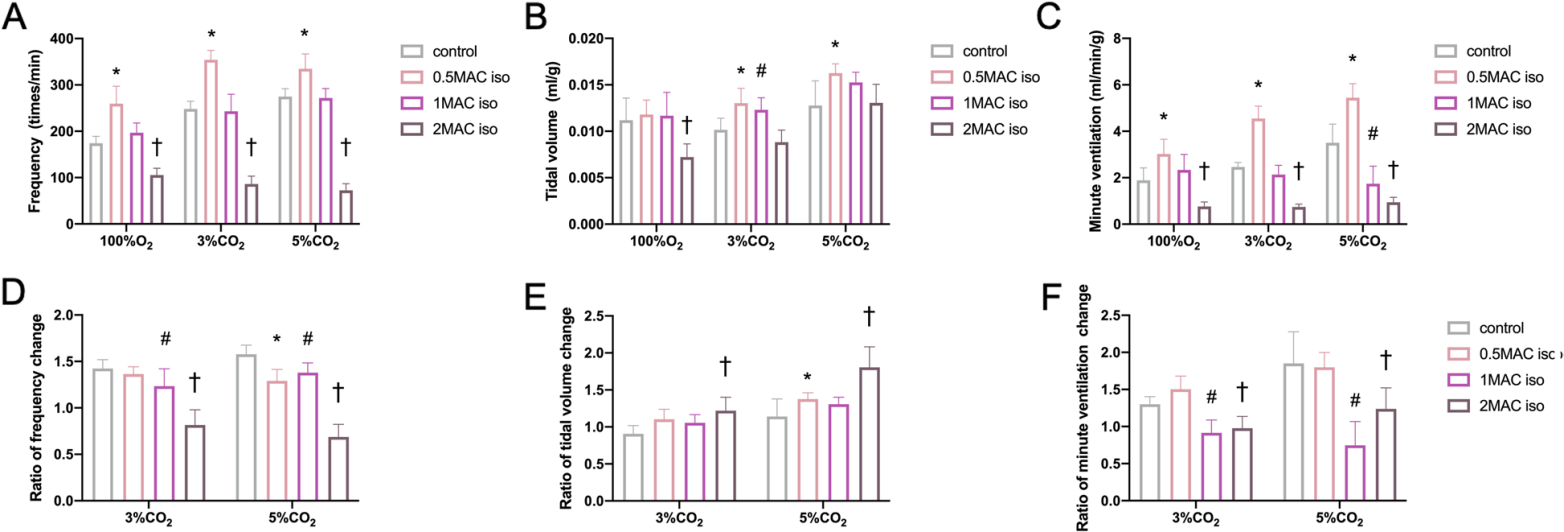
Effect of isoflurane on respiratory function in a dose-dependent manner in vivo. **A-C** Changes in respiratory frequency (**A**), tidal volume (**B**), and minute ventilation (**C**) in the control group and with three doses of sevoflurane (0.5 MAC, 1 MAC, and 2 MAC) are depicted. **D-F** Carbon dioxide induced change ratios in respiratory frequency (**D**), tidal volume (**E**), and minute ventilation (**F**) in the control group and with three doses of sevoflurane (0.5 MAC, 1 MAC, and 2 MAC) are depicted. Data are expressed as mean ± standard deviation. *, #, † indicate that there were significant differences compared with control in 0.5 MAC, 1 MAC, and 2 MAC groups, respectively, by two-way ANOVA (*P* < 0.05)

To compare if these two inhalational anesthetics affected breathing differently, we analyzed respiratory parameters during the period when mice were stable (at 10–12 min) during the 30-min recording. Both sevoflurane and isoflurane increased the respiratory output at concentrations of 0.5 MAC and 1 MAC (Fig. 2 A-F, Fig. 3 A-F). Specifically, at concentrations of 0.5 MAC, sevoflurane and isoflurane both increased FR from 165.57 ± 10.64 to 245.44 ± 31.88 breaths/min (Fig. 2 A, *P* < 0.001) and 174.29 ± 15.02 to 259.41 ± 37.62 breaths/min in 100% O_2_, respectively (Fig. 3 A, *P* < 0.001). Similarly, mice exposed to 0.5 MAC of sevoflurane and isoflurane exhibited increased ventilation under hypercapnia, as follows: sevoflurane: 3% CO_2_ vs. 100% O_2_ produced P values of 0.006, 0.950, and < 0.001 for FR, TV, and MV, respectively, and 5% CO_2_ vs. 100% O_2_ produced P values of 0.001, 0.027, and 0.403 for FR, TV, and MV, respectively; isoflurane: 3% CO_2_ vs*.* 100% O_2_ produced P values of < 0.001, 0.431, and 0.001 for FR, TV, and MV, respectively, and 5% CO_2_ vs. 100% O_2_ produced P values of 0.004, < 0.001, and < 0.001 for FR, TV, and MV, respectively. FR responses to increased CO_2_ were all diminished with administration of 2 MAC sevoflurane (Fig. 2 D, *P* < 0.001 in both 3%CO_2_ and 5%CO_2_ conditions) and isoflurane (Fig. 3 D, *P* < 0.001 in both 3%CO_2_ and 5%CO_2_ conditions) but the TV responses were all increased in 2 MAC sevoflurane (Fig. 2 E, *P* = 0.003 in 3%CO_2_ and *P* = 0.004 in 5%CO_2_ conditions) and isoflurane (Fig. 3 E, *P* < 0.001 in both 3%CO_2_ and 5%CO_2_ conditions).

### Propofol causes significant respiratory depression compared with etomidate

**Fig. 4:**
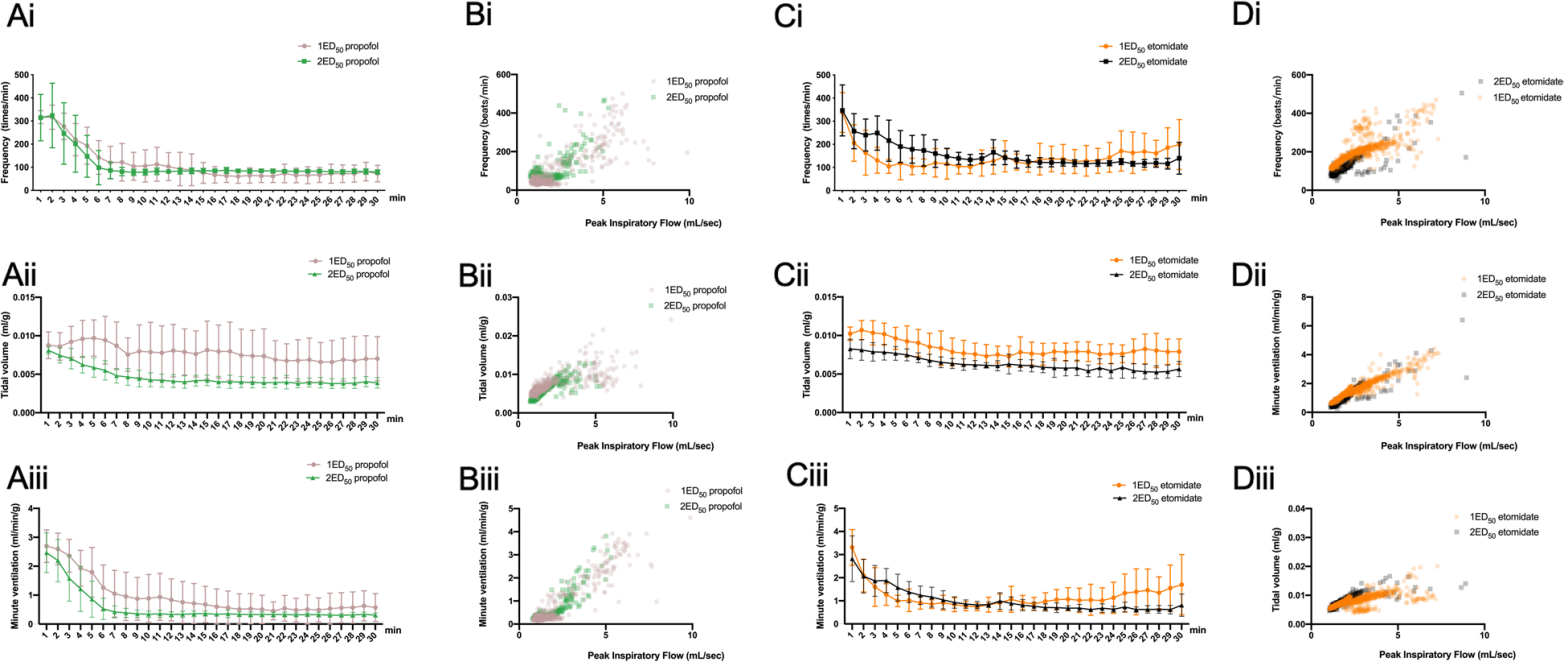
Effect of intravenous anesthetic on respiratory responses in vivo. A Time-course study of 1 median effective dose (ED_50_) and 2 ED_50_ propofol on respiratory frequency (Ai), tidal volume (Aii), and minute ventilation (Aiii) with 100% oxygen in vivo. B Scatterplot of 1 ED_50_ and 2 ED_50_ propofol on respiratory frequency (Bi), tidal volume (Bii), and minute ventilation (Biii) versus airflow (ml/sec) for each breath (dot) taken during the 30-min recording. C Time-course study of 1 ED_50_ and 2 ED_50_ etomidate on respiratory frequency (Ci), tidal volume (Cii), and minute ventilation (Ciii) with 100% oxygen in vivo. D Scatterplot of 1 ED_50_ and 2 ED_50_ etomidate on respiratory frequency (Di), tidal volume (Dii), and minute ventilation (Diii) versus airflow (ml/sec) for each breath (dot) taken during the 30-min recording. Data are expressed as mean ± standard deviation

To explore the dose-dependent effect of intravenous anesthetics on respiratory depression, we used two different concentrations of propofol and etomidate (1 ED_50_ and 2 ED_50_, Fig. 4 A-D). We found that compared with 1 ED_50_, propofol depressed TV in the group exposed to 2 ED_50_ (*P* = 0.004) (Fig. 4 Aii). Etomidate also depressed TV at a higher dose (*P* < 0.001) (Fig. 4 Cii). Scatter plot (tidal volume vs. airflow) also showed that compared to 1 ED_50_, breathing in 2 ED_50_ propofol group became slow and less forceful (Fig. 4 Bii).

**Fig. 5:**
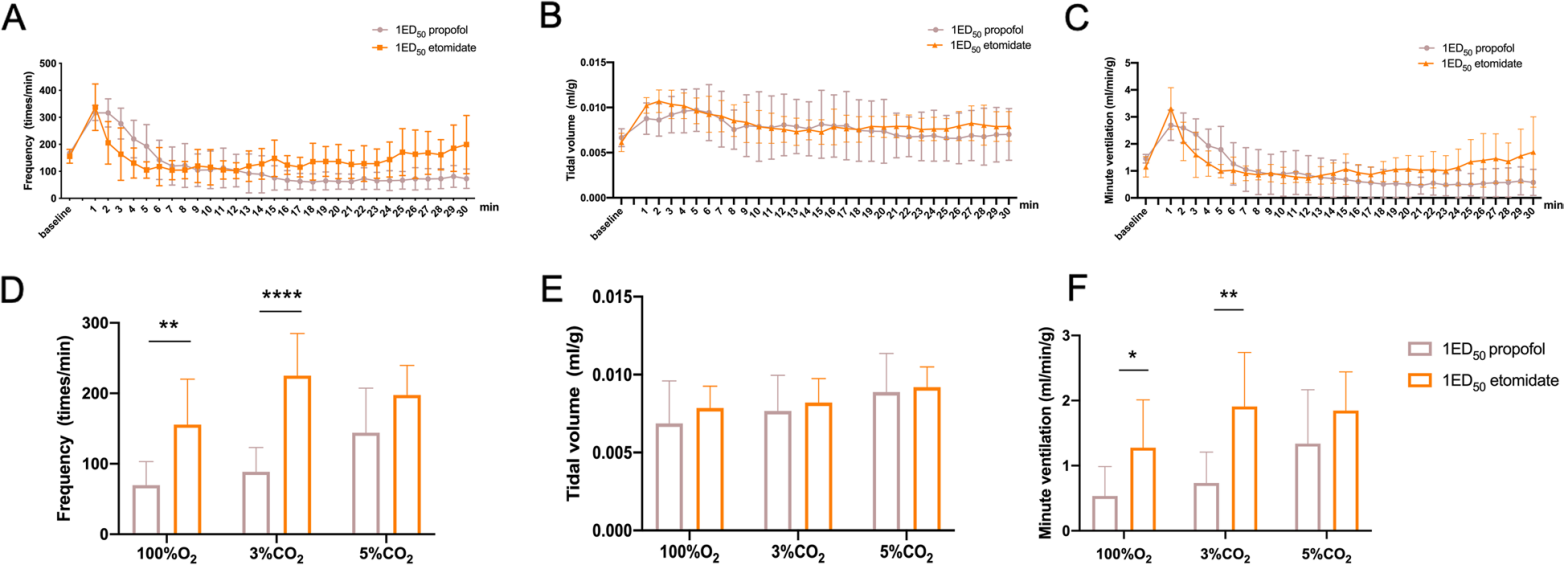
**A**-**C** Time-course study of 1 ED_50_ propofol and etomidate on respiratory frequency (**A**), tidal volume (**B**), and minute ventilation (**C**) with 100% oxygen in vivo. **D-F** Summary data show respiratory frequency (**D**), tidal volume (**E**), and minute ventilation (**F**) responses with 1 ED_50_ propofol and etomidate with graded increases in carbon dioxide (balanced with oxygen). Data are expressed as mean ± standard deviation. **P < 0.01, ***P < 0.001 by two-way ANOVA between 1 ED_50_ propofol and etomidate groups

In mice exposed to 1 ED_50_ of propofol and etomidate, a time-course study revealed that propofol progressively reduced FR, TV, and MV and stabilized after 20 min during the 30-min exposure (Fig. 5 A, B, C). However, there was no further respiratory depression after respiration decreased to a relatively stable level (after 10 min) in the group that received 1 ED_50_ etomidate (Fig. 5 A, B, C). Compared to propofol, etomidate causes less respiratory depression in FR (Fig. 5. D) and MV (Fig. 5 F). In addition, mice that received 1 ED_50_ etomidate showed a diminished CO_2_-dependent increase in FR, TV, and MV. However, mice that received 1 ED_50_ propofol showed a significant increase in FR from 100% O_2_ to 5% CO_2_ (Fig. 5. D, *P* = 0.023), but not TV (Fig. 5. E, *P* = 0.058) or MV (Fig. 5 F, *P* = 0.067).

### Inhalational anesthetics cause less respiratory depression compared with intravenous anesthetics

**Fig. 6:**
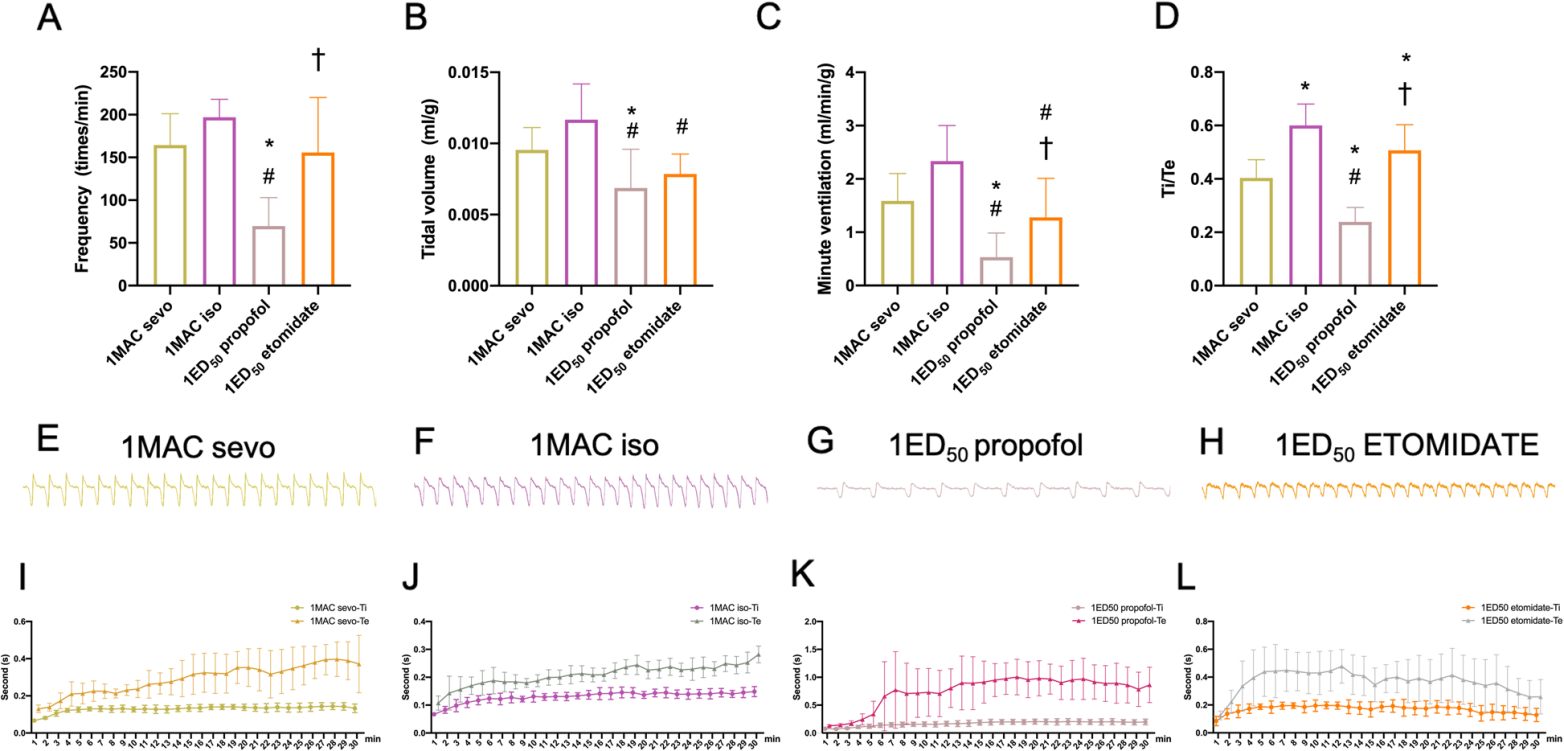
**A**–**D** Summary data show respiratory frequency (**A**), tidal volume (**B**), minute ventilation (**C**), and inspiratory time (Ti)/expiratory time (Te) ratio (**D**) with 1 MAC sevoflurane, 1 MAC isoflurane, 1 ED_50_ propofol and 1 ED_50_ etomidate groups with 100% oxygen. **E**–**H** Characteristic tracings of four general anesthetics on respiratory parameters in vivo. **I**-**L** Time-course study of 1 MAC sevoflurane (**I**), 1 MAC isoflurane (**J**), 1 ED_50_ propofol (**K**) and 1 ED_50_ etomidate (**L**) on Ti and Te with 100% oxygen in vivo. Data are expressed as mean ± standard deviation. *, #, † indicate that there were significant differences compared with sevoflurane, isoflurane, and propofol groups, respectively, by one-way ANOVA (*P* < 0.05)

Unlike with inhalational anesthetics, mice administered intravenous anesthetics cannot be accommodated in the chamber in the beginning of the experiment. Mice in the propofol and etomidate groups became hyperactive after i.p. injection outside the chamber. Thus, we compared the stable period of each general anesthetics. We found that at equivalent doses (1 MAC or 1 ED_50_), sevoflurane and isoflurane caused less respiratory depression compared with propofol and etomidate (Fig. 6 A- 6 L). Propofol caused significant depression in FR, TV and MV after i.p. injection when compared with sevoflurane and isoflurane (Fig. 6 A-C). In addition, we compared Ti, Te, and Ti/Te ratio among the four anesthetics. All four general anesthetics showed an increase in TE. Among them, propofol decreased the Ti/Te ratio (*P* < 0.001 when compared with sevoflurane isoflurane and etomidate groups, Fig. 6D) and increased Te from 0.13 ± 0.01 s to 1.00 ± 0.31 s (Fig. 6K).

## Discussion

In the present study, we found that sub-anesthetic concentrations of sevoflurane and isoflurane increased FR, TV, and MV. In addition, CO_2_-sensitive respiratory responses were maintained to a greater degree with 0.5 and 1 MAC sevoflurane/isoflurane compared with 2 MAC. Meanwhile, in contrast to the etomidate group, propofol showed a dismissed response to graded increases in CO_2_. A time-course analysis revealed that propofol progressively decreased TI/TE ratio during a 30-min exposure compared with sevoflurane, isoflurane, and etomidate at equivalent doses.

In this study, the respiratory response of adult mice to general anesthetics was measured with WBP. WBP is widely used for precise, non-invasive, quantitative measurement of respiratory parameters in unrestrained conditions without intubation [19, 20]. Adult mice were administered sevoflurane, isoflurane, propofol, and etomidate in vivo at doses of 0.5-, 1.0-, and 2.0-times the MAC or ED_50_ required to induce LORR, which included the most commonly used dose ranges of inhalational and intravenous anesthetics. In addition, only the state of immobile respiratory parameters was observed with a sufficient duration for subsequent data analysis. The effect of general anesthesia on the CO_2_-sensitive respiratory response was also determined. Concentrations of sevoflurane and isoflurane were monitored in real-time using the RGM monitor to prevent drug and CO_2_ accumulation.

General anesthetics modulate ventilation by disturbing central chemoreceptor sensitivity, reducing the ventilatory response to hypercapnia, depressing metabolic ventilatory control, and inhibiting the ventilatory adaptation to hypoxia, even at sedative doses [7, 8]. In the present study, both sevoflurane and isoflurane preserved spontaneous breathing and the ventilatory response to hypercapnia at sub-anesthetic concentrations. However, higher CO_2_ sensitivity was not observed in mice administered 2 MAC sevoflurane/isoflurane. Although multiple sites contribute to the depressive effect of general anesthetics on respiration, the relatively selective maintenance of spontaneous breathing is poorly known. Central CO_2_ chemo-sensitivity in mammals is mainly mediated by Phox2B-expressing neurons of the RTN, which were first known for their CO_2_/pH sensitivity and role in providing central chemoreceptor drive to the respiratory system [21–23]. Their CO_2_ sensitivity is unaffected by pharmacological blockade of the respiratory pattern generator and persists without carotid body input [22]. Volatile anesthetics cause activation of RTN neurons, which serve an important integrative role in maintaining respiratory motor activity under immobilizing anesthetic conditions [24]. Depression by propofol may be attributed to an exclusive effect within the central chemoreflex loop at central chemoreceptors. In contrast to sub-concentrations of inhalational anesthetics, the peripheral chemoreflex loop, when stimulated with CO_2_, remains unaffected by propofol [25].

Previous studies demonstrate that sevoflurane-induced respiratory depression is mediated by medullary respiratory and phrenic motor neurons. γ-Aminobutyric acid type A (GABA_A_) receptors may be involved in sevoflurane-induced respiratory depression within the medulla, but not within the spinal cord [13, 26]. Many neuronal elements within the respiratory system are inhibited by inhalational anesthetics. The mammalian pre-Bötzinger complex is an excitatory network of neurons in the medulla that is critically involved in respiration [27]. The effect of inhalational anesthetics on TASK-like channels plays a major functional role in chemosensory modulation of respiratory rhythm in the pre-Bötzinger complex [28]. However, these reported mechanisms have not yet elucidated the difference between the effects of isoflurane and sevoflurane.

In the present study, propofol displayed more obvious respiratory depression on FR, TV, and MV compared with etomidate and volatile anesthetics. Propofol at 1 ED50 exhibited rapid and significant respiratory depression approximately 3 min after i.p. injection compared with 1 ED50 etomidate in our study. Propofol also induced the greatest decrease in TI/TE ratio among the four general anesthetics. Propofol may cause significant airway obstruction [29]. Sedative doses of propofol cause a phase shift between abdominal and ribcage movements under spontaneous breathing without airway support (like the WBP method in our study), thereby decreasing the contribution of ribcage movement to TV and disturbing arterial oxygen tension [30]. It is likely that GABA_A_ receptor-mediated hyperpolarization of neurons serves as the neuronal basis of propofol-induced respiratory depression in *vivo* [31, 32]. Our study provides new insight into the effect of general anesthetics on respiratory behavior, but further study is needed to uncover the underlying mechanisms. One limitation of our study is that we did not include other types of general anesthetic and analgesics, such as ketamine and opioids.

## Conclusion

In conclusion, the present study systematically investigated modulation of respiratory function by general anesthetics and revealed that sevoflurane and isoflurane increase respiratory parameters, even at sub-anesthetic concentrations. Propofol and etomidate depressed TV, but not FR, at higher doses. In addition, propofol induced the greatest decrease in TI/TE ratio among the four general anesthetics. These results suggest that in cases requiring maintenance of spontaneous respiration, sevoflurane or isoflurane may be a better choice than propofol or etomidate.

## Data Availability

The datasets used and/or analysed during the current study are available from the corresponding author (Yu Li) on reasonable request.

## Abbreviations

CO_2_: Carbon dioxide
ED50: Median effective dose
FR: Respiratory frequency
GABA_A_: γ-Aminobutyric acid type A
LORR: Loss of the righting reflex
MAC: Minimum alveolar concentration
MV: Minute ventilation
O_2_: Oxygen
PaCO_2_: Partial pressure of CO_2_ in artery
RTN: Retrotrapezoid nucleus
Ti: Inspiratory time
Te: Expiratory time
TV: Tidal volume
WBP: Whole-body plethysmography

## References

[1] Vutskits L, Xie Z (2016). Lasting impact of general anaesthesia on the brain: mechanisms and relevance. Nat Rev Neurosci.

[2] Global, regional, and national disability-adjusted life-years (DALYs) for 333 diseases and injuries and healthy life expectancy (HALE) for 195 countries and territories, 1990–2016: a systematic analysis for the Global Burden of Disease Study 2016. Lancet. 2017;390(10100):1260–344. 10.1016/S0140-6736(17)32130-X PMC5605707 28919118

[3] Johnston KD, Rai MR (2013). Conscious sedation for awake fibreoptic intubation: a review of the literature. Can J Anaesth.

[4] Levitt ES, Abdala AP, Paton JF (2015). μ opioid receptor activation hyperpolarizes respiratory-controlling Kölliker-Fuse neurons and suppresses post-inspiratory drive. J Physiol.

[5] Varga AG, Reid BT, Kieffer BL (2020). Differential impact of two critical respiratory centers in opioid-induced respiratory depression in awake mice. J Physiol.

[6] Eger EI, Koblin DD, Harris RA (1997). Hypothesis: inhaled anesthetics produce immobility and amnesia by different mechanisms at different sites. Anesth Analg.

[7] Nieuwenhuijs D, Sarton E, Teppema L (2000). Propofol for monitored anesthesia care: implications on hypoxic control of cardiorespiratory responses. Anesthesiology.

[8] Nieuwenhuijs D, Sarton E, Teppema LJ (2001). Respiratory sites of action of propofol: absence of depression of peripheral chemoreflex loop by low-dose propofol. Anesthesiology.

[9] Hsu YW, Cortinez LI, Robertson KM (2004). Dexmedetomidine pharmacodynamics: part I: crossover comparison of the respiratory effects of dexmedetomidine and remifentanil in healthy volunteers. Anesthesiology.

[10] Doi M, Ikeda K (1987). Respiratory effects of sevoflurane. Anesth Analg.

[11] Fee JP, Thompson GH (1997). Comparative tolerability profiles of the inhaled anaesthetics. Drug Saf.

[12] Hatch DJ (1999). New inhalation agents in paediatric anaesthesia. Br J Anaesth.

[13] Kuribayashi J, Sakuraba S, Kashiwagi M (2008). Neural mechanisms of sevoflurane-induced respiratory depression in newborn rats. Anesthesiology.

[14] Stuth EA, Stucke AG, Zuperku EJ (2012). Effects of anesthetics, sedatives, and opioids on ventilatory control. Compr Physiol.

[15] Onimaru H, Homma I (2003). A novel functional neuron group for respiratory rhythm generation in the ventral medulla. J Neurosci.

[16] Calverley RK, Smith NT, Jones CW (1978). Ventilatory and cardiovascular effects of enflurane anesthesia during spontaneous ventilation in man. Anesth Analg.

[17] Zhou C, Liang P, Liu J (2015). HCN1 Channels Contribute to the Effects of Amnesia and Hypnosis but not Immobility of Volatile Anesthetics. Anesth Analg.

[18] Nikaido Y, Furukawa T, Shimoyama S (2017). Propofol Anesthesia Is Reduced in Phospholipase C-Related Inactive Protein Type-1 Knockout Mice. J Pharmacol Exp Ther.

[19] Bastianini S, Alvente S, Berteotti C (2017). Accurate discrimination of the wake-sleep states of mice using non-invasive whole-body plethysmography. Sci Rep.

[20] Hill R, Santhakumar R, Dewey W (2020). Fentanyl depression of respiration: comparison with heroin and morphine. Br J Pharmacol.

[21] Feldman JL, Mitchell GS, Nattie EE (2003). Breathing: rhythmicity, plasticity, chemosensitivity. Annu Rev Neurosci.

[22] Mulkey DK, Stornetta RL, Weston MC (2004). Respiratory control by ventral surface chemoreceptor neurons in rats. Nat Neurosci.

[23] Guyenet PG, Bayliss DA, Stornetta RL, Fortuna MG, Abbott SB, DePuy SD (2009). Retrotrapezoid nucleus, respiratory chemosensitivity and breathing automaticity. Respir Physiol Neurobiol.

[24] Lazarenko RM, Fortuna MG, Shi Y (2010). Anesthetic activation of central respiratory chemoreceptor neurons involves inhibition of a THIK-1-like background K(+) current. J Neurosci.

[25] Kumar NN, Velic A, Soliz J (2015). PHYSIOLOGY. Regulation of breathing by CO_2_ requires the proton-activated receptor GPR4 in retrotrapezoid nucleus neurons. Science.

[26] Umezawa N, Arisaka H, Sakuraba S (2015). Orexin-B antagonized respiratory depression induced by sevoflurane, propofol, and remifentanil in isolated brainstem-spinal cords of neonatal rats. Respir Physiol Neurobiol.

[27] Smith JC, Butera RJ, Koshiya N (2000). Respiratory rhythm generation in neonatal and adult mammals: the hybrid pacemaker-network model. Respir Physiol.

[28] Koizumi H, Smerin SE, Yamanishi T (2010). TASK channels contribute to the K+-dominated leak current regulating respiratory rhythm generation in vitro. J Neurosci.

[29] Shin HJ, Kim EY, Hwang JW (2018). Comparison of upper airway patency in patients with mild obstructive sleep apnea during dexmedetomidine or propofol sedation: a prospective, randomized, controlled trial. BMC Anesthesiol.

[30] Yamakage M, Kamada Y, Toriyabe M (1999). Changes in respiratory pattern and arterial blood gases during sedation with propofol or midazolam in spinal anesthesia. J Clin Anesth.

[31] Kashiwagi M, Okada Y, Kuwana S (2004). A neuronal mechanism of propofol-induced central respiratory depression in newborn rats. Anesth Analg.

[32] Zeller A, Arras M, Lazaris A (2005). Distinct molecular targets for the central respiratory and cardiac actions of the general anesthetics etomidate and propofol. FASEB J.

